# Same-day versus delayed simulation imaging after placement of a perirectal hydrogel spacer for prostate radiotherapy

**DOI:** 10.3389/fonc.2023.1236113

**Published:** 2023-07-14

**Authors:** Elisha Fredman, Miriam Weinstock-Sabbah, Oded Icht, Assaf Moore, Tzippora Shochet, Dror Limon, Dimitri Bragilovski

**Affiliations:** ^1^ Department of Radiation Oncology, Davidoff Cancer Center, Rabin Medical Center, Petah Tikvah, Israel; ^2^ Department of Biostatistics, Rabin Medical Center, Petah Tikvah, Israel

**Keywords:** prostate, radiation, hydrogel, SpaceOAR™, simulation, rectal toxicity

## Abstract

**Introduction:**

Placement of a perirectal hydrogel spacer has been demonstrated to reduce the risk of rectal toxicity from prostate radiation. Practices vary regarding the timing of CT simulation after hydrogel placement, and the ideal schedule remains unknown.

**Methods:**

Thirty patients with localized prostate adenocarcinoma underwent transrectal ultrasound-guided placement of an iodinated SpaceOAR™ hydrogel prior to radiotherapy. Per evolving practice, 15 completed same-day simulation and 15 returned for simulation 1–2 weeks later. Hydrogel volume, perirectal distance, air-void volume, and rectal dosimetry per NRG GU005 were compared between CT simulation, 1st fraction Cone-Beam-CT (CBCT), and final CBCT.

**Results:**

CT simulation occurred 8.8 ± 2.4 days after placement in the delayed group, with no significant difference in the interval between simulation and 1st fraction between groups (*p* = 0.165). Greater observed de-creases in hydrogel volume (0.57 cc *vs.* 0.04 cc, *p* = 0.0002), and perirectal distance at both mid-gland (1.32 mm *vs.* 0.17 mm) and tallest point (2.40 mm *vs.* 0.04 mm) were seen on 1st-fraction CBCT in the same-day group (*p* = 0.0039; *p* = 0.0002). Per dosimetry recalculated on 1st fraction CBCT, five (D3 cc and D50%) versus one (D50%) rectal dose parameters were exceeded in the same-day and delayed groups, respectively, and 10 versus one parameters had a relative increase of ≥ 20%.

**Conclusion:**

Due to the evolving anatomic changes in the days following hydrogel placement, same-day simulation scanning may introduce unintended variability in rectal dosimetry at the time of prostate radiotherapy.

## 1 Introduction

Multiple technological innovations have been developed in recent years to help reduce the risk of acute and late rectal toxicity from prostate radiotherapy. Among them is the advent of perirectal hydrogel spacers, which are transperineally injected in the potential fat space between the posterior prostate and anterior rectal wall, thereby displacing the rectum away from the high-dose radiation field (1, 2). A quick and overall low-risk procedure, prospective trials have demonstrated the clinical benefits of perirectal spacing with hydrogel (3–5).

As with the introduction of any new intervention into a workflow, the ideal timing between hydrogel placement and the next step in the planning process, CT simulation, remains uncertain. Practices vary, ranging from simulation on the same day as the hydrogel procedure, to a delay of days or even weeks (6). The rationale for a same-day simulation is to consolidate the treatment planning process for optimized efficiency and reduced cost. A potential disadvantage of this approach, however, is that the transperineal insertion of the hydrogel may cause local inflammation with associated edema, bleeding, and the introduction of air, all of which will typically resolve during the time interval before the first treatment (7). Such changes could unintentionally alter the relative positioning of the rectum and its proximity to the prostatic target as seen on pre-treatment cone beam CT (CBCT), resulting in unforeseen radiation exposure.

In order to better characterize these potential changes, we analyzed the variation in hydrogel volume, prostate-to-rectum spacing, presence of air-voids, and rectal dosimetry, between CT simulation, 1st-fraction CBCT, and last-fraction CBCT, and compared differences between same-day versus delayed CT simulation.

## 2 Materials and methods

Institutional IRB approval was granted for this retrospective review of a prospectively collected cohort. Patients with localized T1c–T2c adenocarcinoma of the prostate underwent placement of an iodinated hydrogel spacer (SpaceOAR Vue™, Boston Scientific, Watertown MA) in anticipation of definitive radiotherapy. As department procedural workflow for prostate radiation planning was changing, the final 15 patients undergoing same-day simulation were analyzed in comparison with the first 15 for whom simulation was performed the following week.

Hydrogels were inserted with an aseptic transperineal technique under transrectal ultrasound (TRUS) guidance in the department of radiation oncology by the treating physician, certified in hydrogel placement. All patients were prepped for the procedure with 1 week of a daily laxative, an enema on the morning of the procedure, and 3 days of prophylactic Bactrim DS twice daily starting on the day of the procedure. Due to changing practice, the same-day simulation group was given conscious sedation and the delayed simulation group received local anesthesia of 2% xylocaine with epinephrine. The delayed group also underwent intraprostatic insertion of three gold fiducial markers during the procedure.

CT simulations (Toshiba Aquilion RT) were performed either within 3 hr of the procedure or 7–13 days later, with a comfortably full bladder and empty rectum, using 2-mm CT slices, and fused with a planning 3-tesla T2-MRI (Siemens Magnetom Vida) with 1-mm slices, performed within 0–2 days of the CT. The CTV was defined as the prostate (based on MRI and CT) and either the proximal 2 cm or the entire seminal vesicle. A 5-mm isotropic expansion with 3-mm posterior expansion (for stereotactic radiotherapy, SABR) or 7-mm and 5-mm posterior expansion (hypofractionation) comprised the PTV. Rectum was contoured as the outermost wall of the organ as seen on non-contrast planning CT. The hydrogel was defined by the readily visible hyperdense-appearing mass between the prostate and rectum (due to the bound iodine content in SpaceOAR Vue™) including any fluid or air substance contained within the borders of the gel. Contours were independently reviewed by a second senior radiation oncologist and dosimetrist. All treatments were planned with a VMAT technique using 6–10 MV FFF beams to a dose of 35, 37.5, or 40 Gy in five fractions for SABR or 60 Gy in 20 fractions. Prior to each fraction, an MV CBCT of the same slice thickness was performed and aligned to the planning CT.

Upon completion of radiation, hydrogels and rectal volumes were re-contoured on the 1st-fraction and final fraction CBCTs (Varian Truebeam, matched onboard imaging). Simulation CTs were performed using a pre-set standard soft-tissue window while CBCTs were windowed to yield the greatest degree of hydrogel contrast. Hydrogel stability over each of the three scans was assessed based on software-generated volume calculation (Eclipse, Varian Medical Systems, Palo Alto CA), and the measured height of the hydrogel at mid-gland (halfway between superior base and inferior apex slices in sagittal plane) center (midpoint of lateral extent in axial planes) and at its tallest mid-gland center point (along same central axes). Hypodense signal voids consistent with air within the hydrogel were contoured and total collective volume across scans recorded. Volumetric constraints as outlined in the ongoing clinical trial NRG GU005 (8) were utilized to assess rectal dosimetry and relative percentiles compared across time points.

Mean differences between groups were evaluated by Student’s t-test and Wilcoxon rank sum. A meaningful relative dosimetric change between time points was defined as either a parameter changing from meeting to exceeding any of the five constraints, or a ≥ 20% difference between rectal constraint values between time points.

## 3 Results

Thirty patients in total were assessed, 15 each in the same-day and delayed arms, respectively, with balanced patient characteristics (**Table 1**). In both arms, 13 patients had intermediate-risk disease and two had high-risk disease. Mean prostate volume in the same-day and delayed groups was 48.19 cc (19.73–80.71 cc) and 55.28 cc (34.56–97.62 cc), respectively (*p* = 0.418). Thirteen of 15 patients in each group were treated with SABR in five fractions.

**Table 1:** Patient characteristics.

Parameter	Same day (*n* = 15)	Delayed (*n* = 15)
Mean volume (cc)	48.19 (19.73–80.71)	55.28 (34.56–97.62)
Risk group		
Intermediate High	13 (FIR-10; UIR-3) 2	13 (FIR-11; UIR-2) 2
Radiation delivery		
SABR Hypofractionated	13 2	13 2
Dose/fractions		
35/5 37.5/5 40/5 60/20	3 7 3 2	4 3 6 2

FIR, favorable intermediate risk; UIR, unfavorable intermediate risk.

CT simulation occurred on average 8.8 ± 2.4 days (range: 7–13) after hydrogel placement in the delayed group, and 1st-fraction of radiation occurred 24.2 ± 2.0 and 25.7 ± 2.9 days after simulation in the same-day and delayed groups, respectively (*p* = 0.165). There were no reported complications from the hydrogel placement or unforeseen patient-related delays in treatment start.

A greater mean relative change in hydrogel volume from simulation to 1st-fraction CBCT was observed in the same-day group of 0.57cc (12.23 cc to 11.66 cc) compared with 0.04cc (9.74 cc to 9.70 cc) in the delayed group (*p* = 0.0002), representing a mean percent decrease of 5.49% versus 0.04%, respectively (*p* = 0.0002) (**Table 2**).

**Table 2:** Changes in hydrogel parameters between CT simulation and 1st-fraction CBCT.

	Same day (*n* = 15)			Delayed (*n* = 15)		
Parameter	CT Sim	CBCT #1	Change (%)	CT Sim	CBCT #1	Change (%)
Hydrogel vol. (cc)	12.23 ± 3.91	11.66 ± 4.40	5.49	9.74 ± 1.22	9.70 ± 1.20	0.04
Distance (cm)						
Midgland Tallest	1.11 1.42	0.98 1.18	11.71 17.04	1.15 1.41	1.13 1.40	1.74 0.71
Air-void vol. (cc)	1.00	0.00	100%	0.00	0.00	0.00

**Figure 1:**
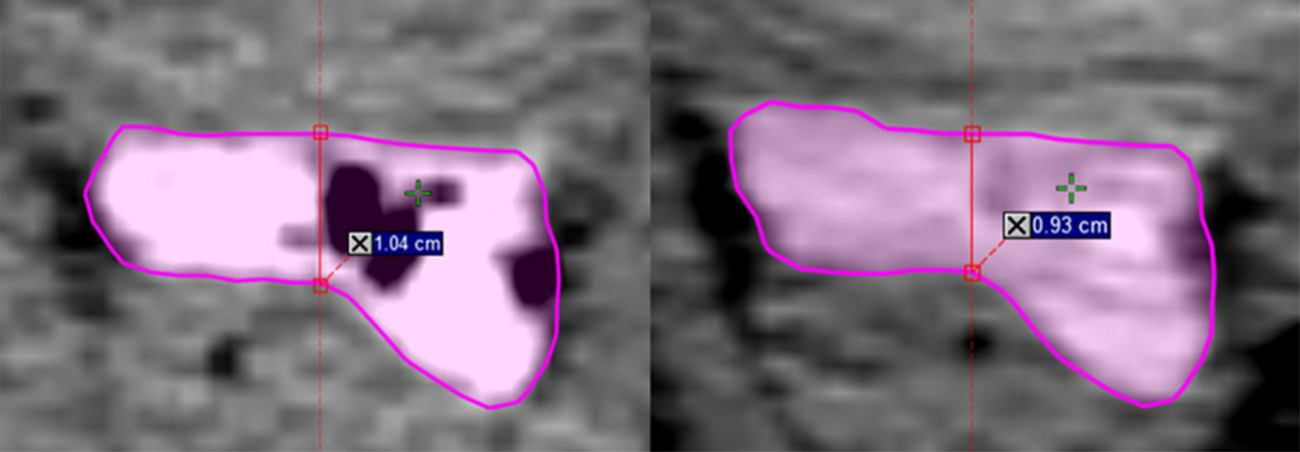
Change in perirectal distance at center mid-gland between same-day CT simulation (left) and 1st fraction CBCT (right).

Perirectal distance as measured on CT simulation versus 1st fraction CBCT also decreased more substantially in the same-day group, with a mean decrease between time points of 1.32 mm (11.71%) versus 0.17 mm (1.74%) at mid-gland, and 2.40 mm (17.04%) versus 0.04 mm (0.71%) at the tallest point along the hydrogel (*p* = 0.0039; *p* = 0.0002) (**Figure 1**). No significant differences were appreciated between the 1st and last CBCT for both five-fraction and 20-fraction radiation regimen.

**Figure 2:**
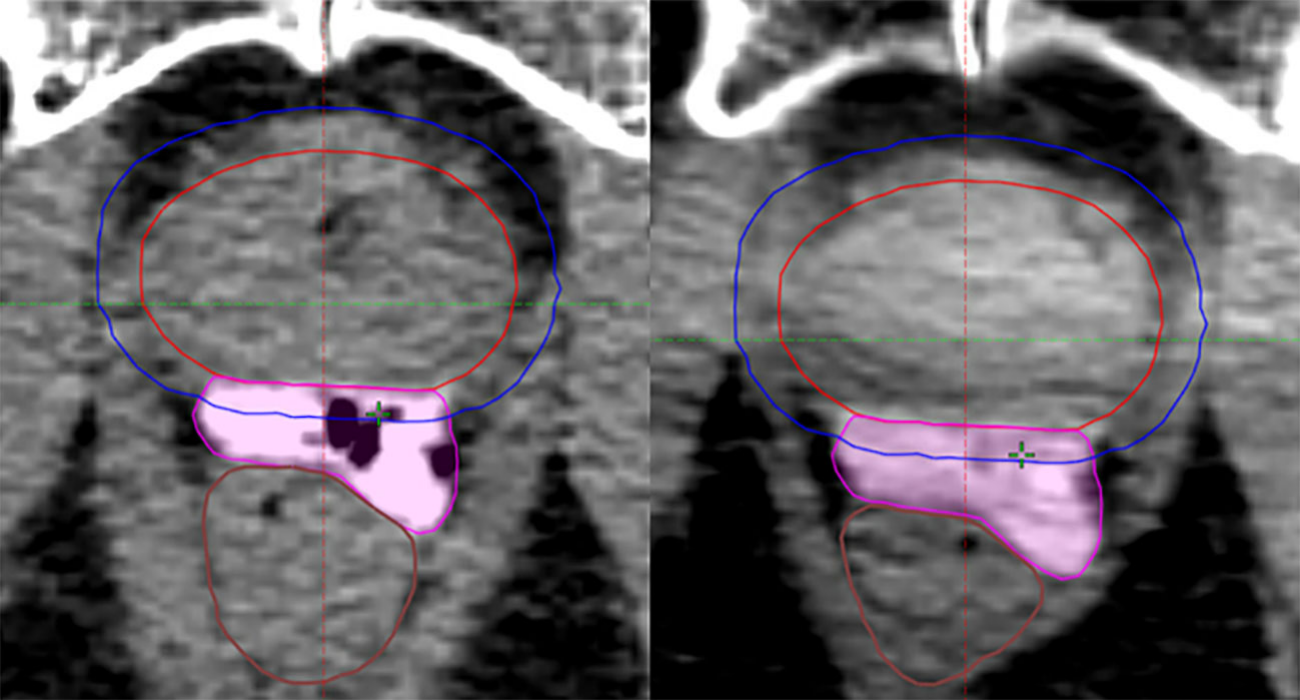
Space-occupying air signals within the hydrogel volume at same-day CT simulation (left), no longer present at 1st fraction CBCT (right).

In the same-day simulation group, a mean of 1.0cc of air signal was measured in the periprostatic space at simulation, with no residual air on the 1st fraction CBCT (**Figure 2**). No air voids were seen in the delayed group.

**Figure 3:**
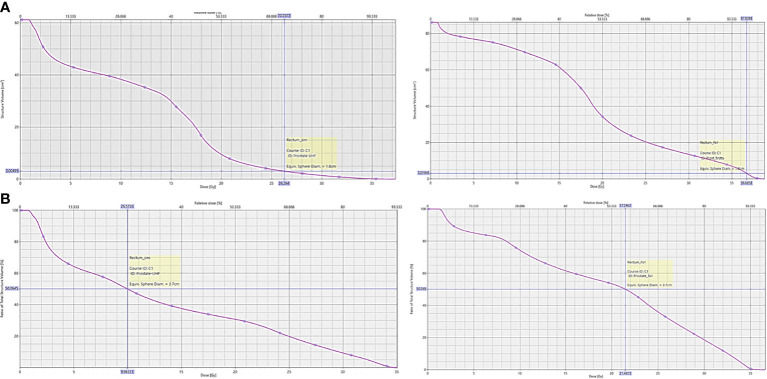
Dose-volume histogram of NRG GU005 rectal dosimetry from planning CT simulation scan versus 1st fraction CBCT from a patient in the same-day CT simulation group. **(A)** Rectal D3cc; **(B)** Rectal D50%.

Upon retrospective contouring of the pelvic structures on the 1st fraction CBCT and recalculating the resulting rectal dosimetry based on NRG GU005, while one of 75 criteria (D50%) was initially exceeded on the CT simulation plan in the same-day group, five rectal dose constraints (D3cc and D50%) were exceeded based on 1st fraction CBCT (**Figure 3**). In comparison, all rectal constraint measures were met based on 1st fraction CBCT in the delayed group. Comparing extent of changes in rectal dosimetry between CT simulation and 1st fraction CBCT, relative increases of at least 10, 12, 15, and 20% were calculated in 12, 11, 11, and 10 measures in the same-day group versus 8, 4, 1, and 1 measures in the delayed group (**Table 3**).

**Table 3:** Changes in rectal dosimetry between CT simulation and 1st-fraction CBCT (NRG GU005).

(Values in %)	Same day CT (*n* = 15)			Delayed CT (*n* = 15)		
Parameter	CT Sim	CBCT #1	Change	CT Sim	CBCT #1	Change
D0.03cc	97.0	89.2	−8.04	89.9	91.2	+1.45
D3cc	80.0	81.5	+1.88	71.8	72.2	+0.56
D10%	71.3	75.3	+5.61	63.2	62.7	−0.79
D20%	61.1	65.1	+6.55	54.2	53.3	−1.66
D50%	38.3	41.4	+8.09	36.9	35.4	−4.07
Mean absolute change			6.03%			1.71%

## 4 Discussion

Holding multiple important variables constant in this single-institution, single-operator, and prospective cohort, we found that measurable material and anatomic changes that occur within the space between the posterior prostate and anterior rectal wall in the days following hydrogel insertion can impact rectal dosimetry. Advantages of our study include the comparative cohort design, consistency of procedural methodology, and utilization of the latest iteration of SpaceOAR Vue™ that can be easily visualized and therefore accurately contoured on CT simulation scans and CBCT.

Our results differ somewhat from other published experiences. Brenneman et al. reported insignificant mean changes of hydrogel size and spacing between CT simulation and a 3- to 4-week verification MRI that did not meaningfully impact rectal dosimetry (9). With a definition of stable as < 10% change in hydrogel volume, they found a range of volume change between −9.6% and 8.6%, and a significance level of *p* = 0.076 for the variation between the two time points. Differences in our study design include a direct comparative cohort, a single operator performing the procedures, as well as direct same-thickness CT-to-CT image fusion versus additional fusions between different imaging modalities, perhaps enabling a greater sensitivity to detect variation that was only partially captured previously.

In their comparison of two perirectal spacing devices, Wolf et al. found a size decrease of only 3% between CT simulation and the end of a 37–40 fraction treatment course (10). In their study, however, simulation was not performed on the day of the procedure, rather days later, allowing time for at least partial resolution of the procedure-induced local inflammatory changes, more similar to the delayed group in our study. Furthermore, precise contouring of the earlier isodense-appearing iteration of SpaceOAR™, especially in the context of post-radiation inflammatory changes, can be challenging and more variable. The iodinated version, SpaceOAR Vue™, used in this analysis has been demonstrated to retain its clear visibility throughout its stability period (11).

Saito et al. compared hydrogel volumes between an MRI obtained on the day of CT simulation and a second MRI at the end of SABR, at a median one-day delay between the hydrogel procedure and simulation (6). Based on contours by two medical physicists, they observed a significant difference between the pre- and post-treatment MRIs, but as the overall mean difference in volume was small, concluded that a single day delay between insertion and simulation was appropriate. This statistical significance emerged despite a range of 1–9 days from procedure to simulation among their 15-patient cohort, none of whom had same-day imaging, further supporting the possibility for unaccounted changes if simulation is performed immediately following hydrogel placement.

Finally, we observed the resolution of air-voids that had been intermixed within and at the margins of the hydrogel structure at the time of same-day CT simulation and no longer present on the 1st fraction CBCT. While the scope of our study did not extend to exploring the potential clinical significance of these findings, changes in the presence of air density along the beam path is a known complicating factor in the consistency of proton radiation fluence (12) and could potentially introduce important variations in dose distribution.

Limitations of our study include a relatively small sample size, although our series represents, to the best of our knowledge, the largest to date directly assessing post-insertion changes in the days following hydrogel spacer placement. An additional limitation is that our analysis was limited to calculated dosimetry without long-term clinical toxicity outcomes. Finally, while they were blinded and reviewed, manual contouring of anatomical structures can be variable with interrater differences, an inherent challenge in this type of study.

The geometry of perirectal hydrogel spacers and presence of procedure-induced local inflammation as seen on same-day CT simulation can evolve in the days following insertion. When compared with CT simulation performed approximately 1 week later, such changes may result in meaningful rectal dosimetric variation at the time of radiotherapy. While both same-day and delayed CT simulation after hydrogel placement are feasible and have merit, the presence of such changes should be considered, and based on the results presented herein, departmental practice was updated to incorporate a planned approximate 1-week delay from hydrogel placement to CT simulation.

## Data availability statement

The raw data supporting the conclusions of this article will be made available by the authors, without undue reservation.

## Ethics statement

The studies involving human participants were reviewed and approved by Helsinki Committee of Rabin Medical Center. Written informed consent for participation was not required for this study in accordance with the national legislation and the institutional requirements.

## Author contributions

All authors contributed equally to this work. EF contributed to study design, procedure performance, study analysis, interpretation of data and drafting and editing of the final manuscript. MW-S contributed to the data collection, analysis, and drafting of the initial manuscript. OI contributed to study design, data collection and analysis, and editing of the manuscript. AM contributed to analysis and manuscript editing. TS contributed to data and statistical analysis, and editing of the final manuscript. DL contributed to study concept design, facilitation of study performance, and editing of manuscript. DB contributed to study design, data analysis, and editing of the final manuscript.
